# Cost–effectiveness of risk-based breast cancer screening programme, China

**DOI:** 10.2471/BLT.18.207944

**Published:** 2018-06-28

**Authors:** Li Sun, Rosa Legood, Zia Sadique, Isabel dos-Santos-Silva, Li Yang

**Affiliations:** a Department of Health Services Research and Policy, London School of Hygiene and Tropical Medicine, London, England.; b Department of Noncommunicable Disease Epidemiology, London School of Hygiene and Tropical Medicine, London, England.; c School of Public Health, Peking University, NO.38 Xueyuan Road, Haidian District, Beijing 100191, China.

## Abstract

**Objective:**

To model the cost–effectiveness of a risk-based breast cancer screening programme in urban China, launched in 2012, compared with no screening.

**Methods:**

We developed a Markov model to estimate the lifetime costs and effects, in terms of quality-adjusted life years (QALYs), of a breast cancer screening programme for high-risk women aged 40–69 years. We derived or adopted age-specific incidence and transition probability data, assuming a natural history progression between the stages of cancer, from other studies. We obtained lifetime direct and indirect treatment costs in 2014 United States dollars (US$) from surveys of breast cancer patients in 37 Chinese hospitals. To calculate QALYs, we derived utility scores from cross-sectional patient surveys. We evaluated incremental cost–effectiveness ratios for various scenarios for comparison with a willingness-to-pay threshold.

**Findings:**

Our baseline model of annual screening yielded an incremental cost–effectiveness ratio of US$ 8253/QALY, lower than the willingness-to-pay threshold of US$ 23 050/QALY. One-way and probabilistic sensitivity analyses demonstrated that the results are robust. In the exploration of various scenarios, screening every 3 years is the most cost–effective with an incremental cost–effectiveness ratio of US$ 6671/QALY. The cost–effectiveness of the screening is reduced if not all diagnosed women seek treatment. Finally, the economic benefit of screening women aged 45–69 years with both ultrasound and mammography, compared with mammography alone, is uncertain.

**Conclusion:**

High-risk population-based breast cancer screening is cost–effective compared with no screening.

## Résumé

**Objectif:**

Modéliser le rapport coût-efficacité d'un programme de dépistage du cancer du sein fondé sur les risques en Chine urbaine, lancé en 2012, comparé à l'absence de dépistage.

**Méthodes:**

Nous avons élaboré un modèle de Markov pour estimer le coût et les effets portant sur la vie entière, au regard des années de vie pondérées par la qualité (QALY), d'un programme de dépistage du cancer du sein chez les femmes à haut risque âgées de 40 à 69 ans. Nous avons tiré ou adopté des données sur l'incidence selon l'âge et la probabilité de transition, dans l'hypothèse d'une évolution naturelle entre les phases du cancer, à partir d'autres études. Nous avons obtenu les coûts directs et indirects de traitement au cours d'une vie en dollars des États-Unis de 2014 ($US) à partir d'enquêtes menées auprès de patientes atteintes du cancer du sein dans 37 hôpitaux chinois. Pour calculer les QALY, nous avons déduit des scores d'utilité à partir d'enquêtes transversales auprès de patientes. Nous avons évalué le rapport coût-efficacité différentiel selon différents scénarios pour établir une comparaison avec un seuil de consentement à payer.

**Résultats:**

Notre modèle de référence de dépistage annuel a donné un rapport coût-efficacité différentiel de 8253 $US/QALY, soit moins que le seuil de consentement à payer de 23 050 $US/QALY. Les analyses à un seul critère de classification et de sensibilité probabiliste ont démontré que les résultats sont fiables. L'examen de différents scénarios a révélé que le dépistage tous les 3 ans présente le meilleur rapport coût-efficacité, avec un rapport coût-efficacité différentiel de 6671 $US/QALY. Le rapport coût-efficacité du dépistage est réduit si toutes les femmes diagnostiquées ne se font pas soigner. Enfin, l'avantage économique lié au dépistage des femmes âgées de 45 à 69 ans par échographie et mammographie, comparé à un dépistage par mammographie uniquement, est incertain.

**Conclusion:**

Le dépistage du cancer du sein dans les populations à haut risque présente un bon rapport coût-efficacité par rapport à l'absence de dépistage.****

## Resumen

**Objetivo:**

Demostrar la rentabilidad de un programa de detección del cáncer de mama basado en el riesgo en las zonas urbanas de China, iniciado en 2012, en comparación con la ausencia de detección.

**Métodos:**

Se desarrolló un modelo Markov para estimar los costes y efectos durante el ciclo vital, en términos de años de vida ajustados por calidad de vida (AVAC), de un programa de detección del cáncer de mama para mujeres con alto riesgo de entre 40 y 69 años de edad. Se obtuvieron o adoptaron datos de la probabilidad de incidencia y transición específicos por edad, sobre la hipótesis de una progresión de la historia natural entre los estadios del cáncer, a partir de otros estudios. Se obtuvieron los costes directos e indirectos del tratamiento vitalicio en dólares estadounidenses (USD) en 2014 a partir de encuestas a pacientes con cáncer de mama en 37 hospitales de China. Para calcular los AVAC, se derivaron las puntuaciones de los servicios públicos de las encuestas transversales a los pacientes. Se evaluaron las relaciones de rentabilidad incrementales para diversos escenarios en comparación con un umbral de la disposición a pagar.

Resultados Nuestro modelo de referencia de detección anual reveló una relación de rentabilidad incremental de 8253 USD/AVAC, por debajo del umbral de disposición a pagar de 23 050 USD/AVAC. Los análisis de sensibilidad unidireccionales y de probabilidad demostraron que los resultados son sólidos. En la exploración de varios escenarios, la detección cada 3 años es la más rentable, con una relación de rentabilidad incremental de 6671 USD/AVAC. La rentabilidad de la detección se reduce si no todas las mujeres diagnosticadas buscan tratamiento. Finalmente, el beneficio económico de la detección en mujeres de 45 a 69 años de edad con ultrasonido y mamografía, en comparación con solo la mamografía, es incierto.

**Conclusión:**

La detección del cáncer de mama basada en la población de alto riesgo es rentable en comparación con la ausencia de detección.

## ملخص

**الغرض:**

وضع نموذج لفعالية التكلفة لبرنامج فحص سرطان الثدي القائم على المخاطر في الصين الحضرية، والذي تم إطلاقه في عام 2012، مقارنة بعدم إجراء فحص.

**الطريقة:**

لقد قمنا بتطوير نموذج ماركوف لتقدير التكاليف والآثار مدى الحياة، بالنسبة سنوات العمر معدلة الجودة (QALYs)، لبرنامج فحص سرطان الثدي للنساء المعرضات لخطر كبير في سن من 40 إلى 69 عاماً. لقد استنبطنا أو انتهجنا بيانات -تعتمد على السن- لاحتمالية الانتقال والحدوث، بافتراض تقدم التاريخ الطبيعي بين مراحل السرطان، من دراسات أخرى. لقد حصلنا على تكاليف العلاج المباشر وغير المباشر طوال العام 2014 بالدولار الأمريكي من مسوحات لمريضات سرطان الثدي في 37 مستشفى صينيًا. لحساب QALY، قمنا باستنتاج تقييمات للمرافق من خلال مسوحات متعددة القطاعات للمرضى. قمنا بتقييم نسب متزايدة لفعالية التكاليف لمختلف السيناريوهات، وذلك للمقارنة مع عتبة الاستعداد للدفع.

**النتائج:**

أسفر نموذجنا الأساسي للفحص السنوي عن نسبة متزايدة لفعالية التكلفة قدرها 8253 دولار أمريكي/QALY، وهي أقل من عتبة الاستعداد للدفع البالغة قيمتها 23050 دولار أمريكي/QALY. أظهرت تحليلات الحساسية أحادية الاتجاه والاحتمالية أن النتائج قوية. ومع استكشاف السيناريوهات المختلفة، فإن الفحص كل 3 سنوات يكون هو الأكثر فعالية من حيث التكلفة، مع نسبة متزايدة لفعالية التكلفة تبلغ 6671 دولارًا أمريكيًا/QALY. تقل فعالية تكلفة الفحص إذا لم تقم كل النساء اللواتي يتم تشخيصهن بالسعي للعلاج. وأخيرًا، فإن الفائدة الاقتصادية لفحص النساء اللواتي تتراوح أعمارهن بين 45 و69 عامًا باستخدام كل من الموجات فوق الصوتية والتصوير الشعاعي للثدي، مقارنةً بالفحص الشعاعي للثدي وحده، غير مؤكدة.

**الاستنتاج:**

إن فحص سرطان الثدي في التجمعات السكانية عالية الخطورة، يعد فعالا من حيث التكلفة مقارنة بعدم إجراء أي فحص.

## 摘要

**目的:**

为 2012 年启动的中国城市基于风险的乳腺癌筛查项目建模进行成本效果分析，与不筛查的情况进行比较。

**方法:**

我们运用马尔可夫模型来估计 40-69 岁高危妇女乳腺癌筛查的终身成本和根据质量调整生命年 (QALYs) 的终身效应。我们从其他研究中推导出或采用了各年龄组发病率和转移概率数据，并假设癌症不同分期间为自然发展病程。我们从中国 37 家医院的乳腺癌患者调查中获得了 2014 年美元 (US$) 的终身直接和间接治疗费用。为了计算 QALYs，我们从横断面患者调查中得出效用分数。我们评估了不同情景下的增量成本效果比，与意愿支付最低阈值进行比较。

**结果:**

我们每年筛查一次的基线模型得到的增量成本效益比为 8253 美元 /QALY，低于 23050 美元 /QALY 的意愿支付最低阈值。单因素和概率敏感性分析表明结果可靠。在探索不同情景时，每三年进行一次筛查最具成本效果，增量成本效果比为 6671 美元 /QALY。如果并非所有被诊断的女性都寻求治疗，筛查的成本效果会降低。最后，与单独使用乳腺钼靶 X 线摄像比较，同时采用超声和乳腺钼靶 X 线检查筛查 45-69 岁女性的经济效益尚不确定。

**结论:**

与不筛查情况相比，高风险人群的乳腺癌筛查具有成本效果。

## Резюме

**Цель:**

Смоделировать экономическую эффективность программы скринингового обследования рака молочной железы на основе оценки риска в городах Китая, которая была запущена в 2012 году, при сравнении с отсутствием скрининга.

**Методы:**

Авторы разработали марковскую модель для оценки затрат на медицинское обслуживание в течение жизни и результатов (с точки зрения количества лет жизни с поправкой на ее качество (QALY)) внедрения программы скринингового обследования рака молочной железы для женщин с высоким риском в возрасте 40–69 лет. Авторы вывели самостоятельно или позаимствовали данные о зависимости частоты возникновения рака от возраста и о вероятности перехода, основываясь на естественной истории прогрессирования между стадиями рака по данным других исследований. В ходе обследований пациентов с раком молочной железы в 37 китайских больницах были получены данные о прямых и косвенных затратах на медицинское обслуживание в течение жизни в долларах США (долл. США) по курсу 2014 года. Чтобы вычислить показатель QALY, авторы получили индексы оценки общего состояния здоровья из перекрестного обследования пациентов. Была проведена оценка инкрементных коэффициентов эффективности затрат для различных сценариев для сравнения с порогом платежеспособности.

**Результаты:**

Созданная авторами базовая модель ежегодного скрининга привела к увеличению коэффициента эффективности затрат в размере 8253 долл. США/QALY, что ниже порога платежеспособности в размере 23 050 долл. США/QALY. Односторонний и вероятностный анализ чувствительности показал, что результаты являются надежными. Исходя из результатов исследования различных сценариев, проведение скринингового обследования через каждые 3 года является наиболее рентабельным с инкрементным коэффициентом эффективности затрат в размере 6671 долл. США/QALY. Экономическая эффективность скринингового обследования снижается, если не все прошедшие диагностику женщины обращаются за лечением. Наконец, экономическая выгодность скринингового обследования женщин в возрасте 45–69 лет с использованием и ультразвука и маммографии по сравнению с результатами при использовании только маммографии является неопределенной.

**Вывод:**

Скрининговое обследование рака молочной железы среди женщин с высоким уровнем риска является экономически эффективным по сравнению с результатами при отсутствии скрининга.****

## Introduction

Breast cancer is the most common cancer among women. Globally, 1.67 million women were diagnosed with breast cancer in 2012, contributing to more than 25% of female cancer incident cases.^1^ The incidence of breast cancer among Chinese women is increasing twice as fast as the global rate.^2^ In China, breast cancer is the most frequently diagnosed cancer and the fifth leading cause of cancer-related deaths.^3^

Breast cancer is a potentially curable disease if diagnosed and treated at an early stage. The Surveillance, Epidemiology, and End Results Programme reported that women diagnosed with breast cancer at an early stage (Stage I or II) have a better prognosis (5-year survival rate, 85–98%) than for advanced breast cancer (5-year survival rate for Stage III or IV, 30–70%).^4^ The strong argument for earlier diagnosis with respect to patient outcome has resulted in the initiation of breast cancer screening programmes in many countries. The aims of such programmes are the early diagnosis and treatment of cancer patients to improve disease outcomes and to reduce mortality.^5^

Although population-based mammography has been widely adopted in high-income countries for more than 30 years,^6^ it is less cost–effective in low- and middle-income countries.^7^ Studies in China,^8^^–^^10^ Ghana^11^ and the Islamic Republic of Iran^12^^,^^13^ have revealed that population-based mammography is not economically attractive. However, a high-risk population-based breast cancer screening programme could contribute to a much higher detection rate^14^^–^^16^ and could therefore be good value for money in low- and middle-income countries.

Experts have recommended ultrasound as an adjunct to mammography among high-risk women.^17^^–^^20^ For patients with dense breasts, non-calcified breast cancers are more likely to be missed by mammography;^21^ ultrasound permits the detection of small, otherwise occult, breast cancer.^22^

**Fig. 1:**
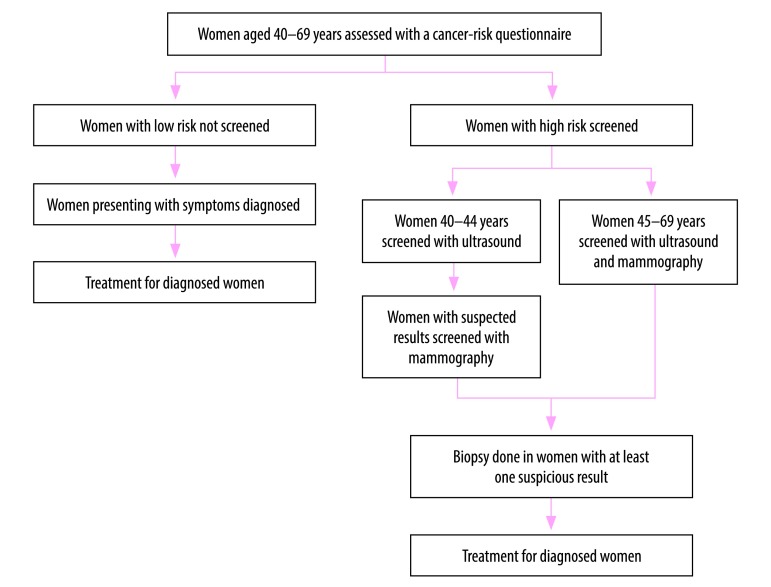
Current risk-based breast cancer screening programme in urban China, launched in 2012

In 2012, the Government of China launched a cancer screening programme in 14 cities to screen common cancers, including breast cancer. Our objective was to provide policy-makers with economic information regarding the cost–effectiveness of breast cancer screening for high-risk women. In this paper, we used a Markov model to compare the lifetime effects, costs and cost–effectiveness of breast cancer screening, versus no screening, using published data from this programme (Fig. 1).

### Methods

#### Screening strategy

To measure the individual risk of breast cancer, health professionals invited women aged 40–69 years to health facilities and used paper-based questionnaires to collect information on individual breast cancer exposure. The health professionals then used the Harvard Cancer Index online tool, now called Your Disease Risk, to process the collected information.^23^^,^^24^ The tool calculates individual cancer scores, by giving risk scores to exposures, including family history, height, age of first period, age of first birth, number of births, age at menopause, use of oral contraceptives, estrogen replacement, Jewish heritage (i.e. higher prevalence of *BRAC1/2* gene mutations) and exposure to ionizing radiation. A total of 198 097 women completed a risk assessment questionnaire during 2012–2013; 17 104 were identified as being at high risk of developing breast cancer.^14^

The programme working group estimated the population average score based on the prevalence of risk factors among the Chinese population, and adjusted according to China’s cancer epidemiology data over 20 years.^14^ The relative risk was obtained by comparing the individual risk score with the population average. Women with a relative risk of > 2 are defined as being at high risk. The programme screens high-risk women aged 40–44 years by ultrasound and the women with suspected results are further examined by mammography. Women with a suspicious mammography result are tested by biopsy for diagnostic confirmation. The programme screens high-risk women aged 45–69 years by both mammography and ultrasound, and suspected results from either method are confirmed with biopsy.

For low-risk women, breast cancer is only diagnosed on presentation of symptoms. Breast cancer patients in the screening arm can be diagnosed while still asymptomatic, that is, at an earlier stage of the disease when prognosis is better.

#### Modelling strategy

Box 1 presents our model assumptions. We adapted a prior natural history Markov model^8^ using the TreeAge software (TreeAge software Inc. Williamstown, United States of America), to inform a long-term decision model. Our model predicted the lifetime costs and quality-adjusted life years (QALYs) of screening and no screening for Chinese urban women with no previous history of breast cancer, from age 40 years to death. We used an annual screening frequency as the baseline, and we explored the scenarios of screening every 3 and 5 years.

Box 1 **Model assumptions for estimating cost–effectiveness ratios of risk-based breast cancer screening programme in urban China**

**Parameters**

- For progression rates between disease stages and relative risk of invasive cancer in ductal carcinoma in situ, we obtained data from other countries and assumed the parameters were applicable to China. We also used disutility score of screening from United Kingdom of Great Britain and Northern Ireland in the baseline analysis. However, we explored the uncertainty in the sensitivity analyses.
- We assumed the risk of developing breast cancer among high-risk women was twice as much as the general population, based on the minimum threshold in Harvard Cancer Index (now called Your Disease Risk).

**Model structure**

- We assumed patients at stage I can progress to stage II, stage III and stage IV. All women can die from non-breast cancer causes during disease progression, but only patients at stage IV can die from breast cancer.
- We assumed all women with suspicious screening findings either with mammography or ultrasound proceeded to diagnostic biopsy. This follows the protocol of the Cancer Screening Programme in Urban China.
- In the base-case analysis, we assumed all breast cancer patients diagnosed by biopsy received treatment. However, because uptake of treatment is uncertain, we explored the scenario where only 70% of detected breast cancers received treatment.

#### Natural history

**Fig. 2:**
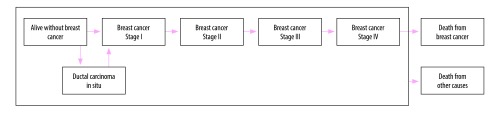
Natural history model for breast cancer progression, China

Notes: The box represents the process of disease progression. We adapted the model from Wong et al.^8^^,^^25^ and Tsokos & Oğuztöreli.^25^

Fig. 2 illustrates the various health states and the potential transitions between them.^8^ Healthy women can transition to ductal carcinoma in situ or stage I cancer, or remain free of cancer. Women with ductal carcinoma in situ are at a higher risk of developing invasive breast cancer (relative risk: 2.02).^4^ Patients at stage I can progress to stage II, stage III and stage IV in turn. All women can die from causes other than breast cancer during disease progression, but only patients at stage IV can die from breast cancer. The state progression transition probabilities used in this analysis are from models described in the literature.^8^^,^^25^

We estimated the probability of symptoms in an unscreened population by calibrating the model as follows. In the non-screening arm, incident cases are only detected on presentation with symptoms; the distribution of incidence cases by stage is therefore a function of the probability of transitions and the probability of symptoms.^26^ We adjusted the probability of symptoms until the distribution of cases presented at each stage was similar to the distribution of reported incidence cases.^3^^,^^27^ Our estimates of transition probabilities are provided in Table 1.

**Table 1:** Parameter values for modelling cost–effectiveness of risk-based breast cancer screening programme launched in 2012 in urban China

Variables	Baseline	Minimum	Maximum	Distribution	Reference/source
**Disease state progression transition probabilities**					
Age-specific incidence, years					
40–44	0.0006100	–	–	–	Chinese Cancer Registry Annual Report^3^
45–49	0.0010056	–	–	–	Chinese Cancer Registry Annual Report^3^
50–54	0.0011650	–	–	–	Chinese Cancer Registry Annual Report^3^
55–59	0.0011179	–	–	–	Chinese Cancer Registry Annual Report^3^
60–64	0.0010458	–	–	–	Chinese Cancer Registry Annual Report^3^
65–69	0.0009782	–	–	–	Chinese Cancer Registry Annual Report^3^
70–74	0.0009912	–	–	–	Chinese Cancer Registry Annual Report^3^
75–79	0.0009067	–	–	–	Chinese Cancer Registry Annual Report^3^
80–84	0.0007803	–	–	–	Chinese Cancer Registry Annual Report^3^
≥ 85	0.0006430	–	–	–	Chinese Cancer Registry Annual Report^3^
Ratio of DCIS incidence to invasive breast cancer incidence	0.12	–	–	–	Lu et al.^28^
RR of invasive cancer from DICS	2.02	–	–	–	SEER Program^4^
Progression rate					
Stage I–Stage II	0.06	–	–	–	Tsokos & Oğuztöreli^25^
Stage II–Stage III	0.11	–	–	–	Tsokos & Oğuztöreli^25^
Stage III–Stage IV	0.15	–	–	–	Tsokos & Oğuztöreli^25^
Stage IV–death	0.23	–	–	–	Wong et al.^8^
Stage-specific probability of symptoms					
Stage I	0.004	–	–	–	Model calibration
Stage II	0.014	–	–	–	Model calibration
Stage III	0.380	–	–	–	Model calibration
Stage IV	0.980	–	–	–	Model calibration
Annual fatality rate after treatment					
Stage I	0.006	–	–	–	Ginsberg et al.^27^
Stage II	0.042	–	–	–	Ginsberg et al.^27^
Stage III	0.093	–	–	–	Ginsberg et al.^27^
Stage IV	0.275	–	–	–	Ginsberg et al.^27^
**Effectiveness of screening**					
Ultrasound followed by mammography if required^a^					
Sensitivity	0.848	0.681	0.949	Beta	Huang et al.^29^
Specificity	0.994	0.990	0.996	Beta	Huang et al.^29^
Ultrasound and mammography^b^					
Sensitivity	0.939	0.798	0.993	Beta	Huang et al.^29^
Specificity	0.980	0.975	0.985	Beta	Huang et al.^29^
**Utility scores**					
Stage I	0.79	0.77	0.80	Log-normal	Shi et al.^30^
Stage II	0.79	0.78	0.80	Log-normal	Shi et al.^30^
Stage III	0.77	0.76	0.79	Log-normal	Shi et al.^30^
Stage IV	0.69	0.65	0.72	Log-normal	Shi et al.^30^
Disutility from false-positive	0.25	0.11	0.34	Log-normal	Peasgood et al.^31^
**Costs, US$**					
Questionnaire	1.6	1.1	2.1	Gamma	Cancer Screening Programme in Urban China^32^
Screening	85.5	59.8	111.1	Gamma	Cancer Screening Programme in Urban China^32^
Biopsy	45.6	31.0	59.3	Gamma	Cancer Screening Programme in Urban China^32^
Treatment costs					
DCIS	2435	1705	3166	Gamma	Li et al.^33^
Stage I	10 067	7047	13 087	Gamma	Liao et al.^34^
Stage II	11 068	7748	14 388	Gamma	Liao et al.^34^
Stage III	12 867	9007	16 727	Gamma	Liao et al.^34^
Stage IV	17 766	12 436	23 096	Gamma	Liao et al.^34^

DCIS: ductal carcinoma in situ; US$: United States dollars.

^a^ For women aged 40–44 years.

^b^ For women aged 45–69 years.

We assumed that all suspected cases proceeded to biopsy and that all diagnosed cases received treatment at baseline. We also explored a scenario of only 70% treatment uptake.

#### Epidemiological and clinical data

We obtained the age-specific invasive breast cancer incidences from the 2012 Chinese Cancer Registry Annual Report.^3^ Since ductal carcinoma in situ incidence is not recorded locally, we estimated the proportion of ductal carcinoma in situ among all breast cancer incidence cases from a Chinese study of 3838 patients.^28^ We calculated age-specific mortalities from other causes by subtracting age-specific breast cancer mortality rates^35^ from the corresponding age-specific all-cause mortality rates.^36^

#### Costs

Data describing the costs of questionnaire, screening (whether ultrasound followed by mammography if required or ultrasound plus mammography, depending on age) and biopsy were available from the screening programme.^32^ We also obtained the treatment costs by stage from the study by the programme working group;^34^ such treatment cost data were estimated from 2746 invasive breast cancer patients from 37 hospitals across 13 provinces in China, comprising direct medical costs, direct non-medical costs and indirect costs. We used the disposable income per capita of Chinese urban residents (22.5 United States dollars (US$) per day)^37^ and productivity loss days to calculate the indirect costs. The Chinese screening programme did not report treatment costs for women with ductal carcinoma in situ, so we estimated these costs from a study of 211 Sichuan Cancer Hospital patients.^33^ All costs are presented at 2014 values. We used the purchasing power parity conversion factor to convert cost values to US$, with US$ 1 equal to 3.51 Chinese yuan.^38^

#### Effectiveness of screening

We used the sensitivity (probability of positive diagnosis if diseased) and specificity (probability of negative diagnosis if not diseased) values from an earlier study^29^ that enrolled 3062 Chinese women (average age, 45 years) at risk of breast cancer. 11 screening modalities were compared, which are different combinations of clinical breast examination, mammography and ultrasound.******We varied the estimates in the sensitivity analyses in case of any variation in diagnostic performance due to the age of the screened population.

#### QALYs

QALY is a measurement that reflects both length of life and health-related quality of life. It is calculated as the product of the utility score of a particular state of health, defined as a dimensionless number between 1 (perfect health) and 0 (death), and the number of years lived. We identified the utility scores for patients at stage I, II, III and IV from a cross-sectional survey conducted as part of the screening programme,^30^ in which breast cancer patients across 13 Chinese provinces completed EuroQol five-dimensional questionnaires.

False-positive results could be argued to undermine quality of life due to psychological distress incurred;^39^ a systematic review estimated a utility decrement (disutility) of 11–34% for false-positive results.^31^ We estimated a loss of 25% at baseline^40^ and explored the uncertainty in the sensitivity analysis.

#### Analysis

In agreement with the China Guidelines for Pharmacoeconomic Evaluations,^41^ we conducted the analysis from a societal perspective. In agreement with these guidelines,^41^ we discounted future costs and future benefits at 3%. We estimated the lifetime costs of screening and its effects in terms of QALY. We calculated the incremental cost–effectiveness ratios, defined as the difference in cost divided by the change in QALY. The willingness-to-pay threshold was estimated to be three times the gross domestic product (GDP) per capita in China in 2014 (US$ 7683).^42^ An incremental cost–effectiveness ratio of less than US$ 23 050/QALY^41^ is therefore an indication that the risk-based breast cancer screening for urban Chinese women aged 40–69 years, compared with no screening, is cost–effective.

To explore the effect of parameter uncertainty, we conducted one-way and probabilistic sensitivity analyses. In the one-way sensitivity analysis, we used the minimum and maximum estimates for effectiveness of screening, utility scores and costs. We varied each parameter individually to assess its impact on overall results. In the probabilistic sensitivity analysis, we varied all variables simultaneously to further explore model uncertainty. The input variables were specified as distributions: costs have a gamma distribution; QALY values follow a log-normal distribution; and sensitivity and specificity of screening follow a beta distribution as suggested in the literature.^43^ By varying input parameters over their respective distributions, we obtained 1000 estimates of incremental costs and incremental effects. We then plotted the cost–effectiveness acceptability curves to show the proportion of simulations for which the intervention was cost–effective at different willingness-to-pay thresholds.

Other scenarios explored included: (i) the impact of screening every 3 years or every 5 years, compared with no screening; (ii) screening every year, but only 70% of the detected cases having access to breast cancer treatment; and (iii) screening women aged 45–69 years every 1, 3 and 5 years via mammography and ultrasound, compared with mammography alone (maintaining the original screening strategy for women aged 40–44 years).

## Results

Our model estimated 43 incident cases of breast cancer per 1000 women over a lifetime; 21 were detected via screening and 22 on presentation with symptoms. Table 2 reports the discounted lifetime costs, QALYs and incremental cost–effectiveness ratios. Overall, the risk-based breast cancer screening yielded higher QALYs compared with no screening (23.0129 QALYs versus 22.9843 QALYs), but was more expensive than no screening (US$ 335.43 versus US$ 99.68). The baseline discounted incremental cost–effectiveness ratio was US$ 8253/QALY, well below the threshold of US$ 23 050/QALY, indicating that the risk-based breast cancer screening programme is cost–effective.

**Table 2:** Modelled cost–effectiveness ratios of risk-based breast cancer screening programme in urban China, 2014

Comparators	Lifetime costs per case (US$)	QALY	Incremental costs (US$)	Difference in QALY	ICER (95% CI)^a^
**Baseline analysis**					
No screening	99.68	22.9843	–	–	–
Annual screening	335.43	23.0129	235.76	0.0286	8 253 (6 170 to 11 483)
**Screening programme variations versus no screening**					
Screening every 3 years	184.67	22.9971	84.99	0.0127	6 671 (5 019 to 9 048)
Screening every 5 years	152.09	22.9919	52.41	0.0076	6 917 (5 157 to 9 416)
Annual screening, but only 70% of detected cases treated	324.17	23.0043	224.49	0.0200	11 223 (8 137 to 17 127)
**Mammography only versus mammography and ultrasound^b^**					
Annual screening	306.41	23.0115	−29.02	−0.0014	21 246 (−172 049 to 168 866)
Screening every 3 years	172.94	22.9960	−11.73	−0.0011	11 000 (−73 330 to 99 983)
Screening every 5 years	145.37	22.9912	−6.72	−0.0007	9 366 (−114 804 to 98 149)

CI: confidence interval; ICER: incremental cost–effectiveness ratio; RR: relative risk; QALY: quality-adjusted life year; US$ United States dollars.

^a^ Discounted at 3%.

^b^ For women aged 45–69 years. Screening regime for women aged 40–44 years remains unchanged.

Note: Some inconsistency arise in some value due to rounding.

**Fig. 3:**
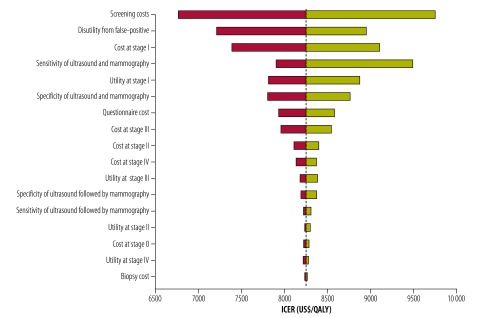
One-way sensitivity analysis of modelled cost–effectiveness of risk-based breast cancer screening programme, urban China, 2014

ICER: incremental cost–effectiveness ratio; QALY: quality-adjusted life year; US$: United States dollars.

Notes: The width of the bars represents the range of ICER when each parameter was varied individually. The vertical dashed line represents incremental cost–effectiveness ratio of US$ 8253/QALY.

**Fig. 4:**
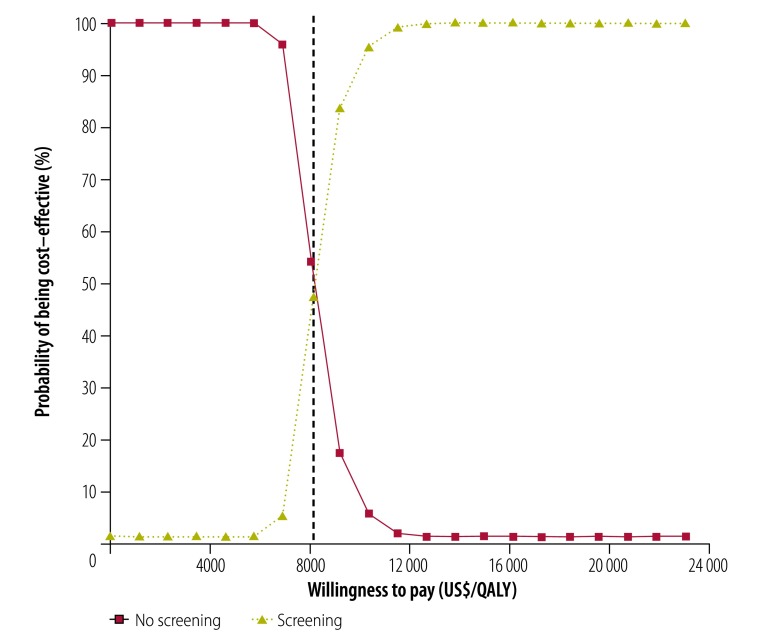
Probabilistic sensitivity analysis of modelled cost–effectiveness of risk-based breast cancer screening programme, urban China, 2014

QALY: quality-adjusted life year; US$: United States dollars.

Note: The vertical dashed line represents incremental cost–effectiveness ratio of US$ 8253/QALY.

The one-way sensitivity analysis (Fig. 3) indicates that the costs, utility scores and effectiveness of screening have little individual influence on the cost–effectiveness of the programme. We found the incremental cost–effectiveness ratios to be lower than the threshold at both the upper and lower limits of these variables. The results of the probabilistic sensitivity analysis (Fig. 4) show that, at the threshold of US$ 23 050/QALY, nearly 100% of the simulations indicate that the risk-based breast cancer screening programme is cost–effective compared with no screening.

In the scenario analysis (Table 2), screening every 3 years and every 5 years achieves an incremental cost–effectiveness ratio of US$ 6671/QALY and US$ 6917/QALY, respectively. A scenario of annual screening, but where only 70% of detected cases are treated, yields a higher incremental cost–effectiveness ratio of US$ 11 223/QALY, which is still lower than the threshold. We also found the scenario of both mammography and ultrasound for women aged 45–69 years, compared with mammography alone, to be cost–effective. However, in the probabilistic sensitivity analysis, the confidence intervals of the incremental cost–effectiveness ratios are very wide: an indication of considerable uncertainty.

## Discussion

The results indicate that compared with no screening, the risk-based breast cancer screening programme is cost–effective. The results prove to be robust in the sensitivity analyses when we varied the estimates for effectiveness of screening, utility scores and costs.

Our finding that high-risk population-based breast screening is cost–effective has implications for breast cancer control in other low- and middle-income countries. Previous studies have reported that population-based mammography screening is not economically attractive in countries, such as the Islamic Republic of Iran and Ghana, with incremental cost–effectiveness ratios of US$ 389 184/QALY^12^ and US$ 12 908/QALY,^11^ respectively. The Chinese screening programme is more likely to be cost–effective than other general population-based screening programmes, since the detection rate in the Chinese programme is higher (16%)^14^ than in general screening programmes (e.g. 3% in the United States of America and 6% in New Zealand).^15^^,^^16^ This finding is consistent with the study comparing risk-based breast cancer screening strategies with general programmes, reporting that risk-based strategies result in greater health benefits for a given cost.^44^

For high-risk women aged 45–69 years, our scenario analysis shows that the benefits of ultrasound in addition to mammography are considerably uncertain. The wide confidence intervals, indicating uncertainty in the incremental cost–effectiveness ratios, do not appear to justify the increased costs. A potential alternative to the current screening strategy could therefore be mammography screening alone for high-risk women aged 45–69 years, instead of both ultrasound and mammography.

Screening every 3 years is the most cost–effective frequency among alternatives. Compared with screening every year, screening every 3 years decreases the total costs significantly, but does not change the effects significantly. The results vindicate the 3-year screening interval for breast cancer in some countries, such as the United Kingdom of Great Britain and Northern Ireland.^5^

Our study explored the impact of access to treatment on the overall results, suggesting that the screening programme is less cost–effective if not all detected cases go on to receive treatment. In China, patients need to pay on average 34% of total medical costs;^45^ this can limit access to medical treatment for some women who have been diagnosed with breast cancer. Some women may also decide not to seek medical treatment if they are not experiencing any pain or do not feel ill;^46^ such delays in the onset of treatment can however lead to a poorer prognosis,^46^ reducing the cost–effectiveness of a screening programme.

As with the previous models,^8^ we adopted the Markov approach in our modelling. While costs and quality of life are provided in the publications by the Chinese screening programme,^30^^,^^32^^,^^34^ no long-term follow-up data are available. We therefore used a mathematical model from age 40 years to death to reflect the differences in costs and effects. We also adopted a prior natural history model, meaning that women free of breast cancer first transition to ductal carcinoma in situ or stage I, followed by the remaining stages in sequence; in contrast, another study^47^ used a model in which it is possible to progress from being free of breast cancer to stage IV. In addition, we calibrated our model to estimate the probability of symptoms by cancer stage, using the distribution of incidence cases reported in the Chinese Cancer Registry Annual Report 2012^3^ in an unscreened population.

Further, we incorporated the decrements in health-related quality of life from false-positive screening results into our model. In this analysis, we used a loss of 25% at baseline and explored the uncertainty (11–34%). However, the utility loss from false–positive results^39^ remains controversial. Although some argue that pathologically elevated levels of distress and anxiety are not apparent,^48^ the relatively small number of studies means that the long-term effects of false-positive breast cancer screening are still unknown.^48^ In this analysis we used estimates from studies based in the United Kingdom of Great Britain and Northern Ireland,^31^^,^^40^ which might bias the cost–effectiveness results of the Chinese screening programme. However, we explored the uncertainty and the results proved to be robust through the sensitivity analyses.

Limitations of our study also include the assumption of high-risk women having a cancer risk index twice that of other women;^23^ the real relative risk among high-risk women in urban China is still unknown. Further, the costs of questionnaires and clinical screening in this study are derived from the cost accounting of the screening programme; other implementation costs such as the identification of eligible women, the administration of risk questionnaires and other ancillary costs were not included. This may lead to an underestimation of costs and subsequently the cost–effectiveness. For progression rates between stages and the relative risk of invasive cancer from ductal carcinoma in situ, we used data from other countries and assumed the parameters were applicable to China. These factors require careful consideration and further research is required to reduce uncertainty.

We used three times the Chinese gross domestic product (GDP) per capita as the willingness-to-pay threshold in our cost–effectiveness analysis. Although GDP-based thresholds are commonly cited,^41^ they have been critized.^49^ Even if estimated accurately, GDP-based cost–effectiveness ratios, or other estimates of willingness to pay, do not provide information on affordability, budget impact or the feasibly of implementation. Although cost–effectiveness ratios are informative in assessing value for money, willingness-to-pay thresholds should therefore not be used alone as a decisions rule for priority setting. Local policy context must also be considered.^49^

In conclusion, our analysis provides economic evidence for the cost–effectiveness of risk-based breast cancer screening in urban China.
